# Improving the diagnosis of cobalamin and related defects by genomic analysis, plus functional and structural assessment of novel variants

**DOI:** 10.1186/s13023-018-0862-y

**Published:** 2018-07-24

**Authors:** Sandra Brasil, Fátima Leal, Ana Vega, Rosa Navarrete, María Jesús Ecay, Lourdes R. Desviat, Casandra Riera, Natàlia Padilla, Xavier de la Cruz, Mari Luz Couce, Elena Martin-Hernández, Ana Morais, Consuelo Pedrón, Luis Peña-Quintana, Miriam Rigoldi, Norma Specola, Isabel Tavares de Almeida, Inmaculada Vives, Raquel Yahyaoui, Pilar Rodríguez-Pombo, Magdalena Ugarte, Celia Pérez-Cerda, Begoña Merinero, Belén Pérez

**Affiliations:** 1 Centro de Diagnóstico de Enfermedades Moleculares, Centro de Biología Molecular, Universidad Autónoma de Madrid, CIBERER, IdiPAZ, Madrid, Spain; 2 Grupo de Bioinformática Translacional Vall d’Hebron Institute of Research (VHIR), Universitat Autònoma de Barcelona, Barcelona, Spain; 3 ICREA, Barcelona, Spain; 4 Hospital Clínico Universitario de Santiago, Santiago de Compostela, CIBERER, Santiago de Compostela, Spain; 5 Hospital 12 de Octubre, Madrid, Spain; 6 Hospital Universitario La Paz, Madrid, Spain; 7 Hospital Universitario Niño Jesús, Madrid, Spain; 8 Hospital Universitario Materno Infantil, CIBEROBN, Universidad de Las Palmas de Gran Canaria, Las Palmas de Gran Canaria, Spain; 9 Center for Rare Disorders, ASST- Monza, Ospedale San Gerardo, Monza, Italy; 10 Unidad de Metabolismo Hospital de Niños de La Plata, La Plata, Argentina; 11 Metabolic Diseases Unit, Lisbon, Portugal; 12 Hospital Virgen de la Arrixaca, Murcia, Spain; 13 Hospital Universitario Regional de Málaga, Instituto de Investigación Biomédica de Málaga (IBIMA), Málaga, Spain

**Keywords:** Cobalamin disorders, Methylmalonic aciduria, Homocystinuria, Massive parallel sequencing

## Abstract

**Background:**

Cellular cobalamin defects are a locus and allelic heterogeneous disorder. The gold standard for coming to genetic diagnoses of cobalamin defects has for some time been gene-by-gene Sanger sequencing of individual DNA fragments. Enzymatic and cellular methods are employed before such sequencing to help in the selection of the gene defects to be sought, but this is time-consuming and laborious. Furthermore some cases remain undiagnosed because no biochemical methods have been available to test for cobalamin absorption and transport defects.

**Results:**

This paper reports the use of massive parallel sequencing of DNA (exome analysis) for the accurate and rapid genetic diagnosis of cobalamin-related defects in a cohort of affected patients. The method was first validated in an initial cohort with different cobalamin defects. Mendelian segregation, the frequency of mutations, and the comprehensive structural and functional analysis of gene variants, identified disease-causing mutations in 12 genes involved in the absorption and synthesis of active cofactors of vitamin B_12_ (22 cases), and in the non-cobalamin metabolism-related genes *ACSF3* (in four biochemically misdiagnosed patients) and *SUCLA2* (in one patient with an unusual presentation)*.* We have identified thirteen new variants all classified as pathogenic according to the ACGM recommendation but four were classified as variant likely pathogenic in *MUT* and *SUCLA2*. Functional and structural analysis provided evidences to classify them as pathogenic variants.

**Conclusions:**

The present findings suggest that the technology used is sufficiently sensitive and specific, and the results it provides sufficiently reproducible, to recommend its use as a second-tier test after the biochemical detection of cobalamin disorder markers in the first days of life. However, for accurate diagnoses to be made, biochemical and functional tests that allow comprehensive clinical phenotyping are also needed.

**Electronic supplementary material:**

The online version of this article (10.1186/s13023-018-0862-y) contains supplementary material, which is available to authorized users.

## Background

Cellular cobalamin problems are caused by nutritional deficiency or genetic defects that affect either the absorption or cellular uptake of the vitamin or the synthesis from it of methylcobalamin (MeCbl) and adenosylcobalamin (AdoCbl). The latter are, respectively, a cofactor of methionine synthase (MS EC_2.1.1.13), which catalyzes the remethylation of homocysteine (Hcys) to methionine in the cytoplasm, and of methylmalonyl-CoA mutase (MUT EC_5.4.99.2), which catalyzes the mitochondrial isomerization of L-methylmalonyl-CoA (MMACoA) to succinyl-CoA. Defects in the synthesis of AdoCbl or the conversion of MMACoA to succinyl-CoA lead to elevated methylmalonic acid (MMA) concentrations, while defects in the synthesis of MeCbl or the remethylation of Hcys to methionine cause elevated Hcys (HC). Defects in the absorption and transport of vitamin B_12_, and in the cytosolic synthesis of the above cofactors, cause methylmalonic aciduria with homocystinuria (MMA&HC) [1, 2].

After its intake, cobalamin is first bound to haptocorrin (encoded by *TCN1*) and then to the intrinsic factor (IF) encoded by *GIF*. To date, only one pathogenic mutation and one functional polymorphism (p.Asp301Tyr in *TCN1*) have been described [3, 4]. Cubilin and amnionless, encoded by *CUBN* and *AMN* respectively, form the cubam dimer which functions as the receptor of IF-Cbl in the ileum [4]. The IF is then degraded and vitamin B_12_ appears in the blood stream associated with transcobalamin II (TCII) [2, 5]. Inherited malabsorption of cobalamin causes haematological and neurological abnormalities that can be fatal [6].

Still bound to TCII, the vitamin enters cells via transcobalamin receptor (TCblR)-mediated endocytosis. This receptor is encoded by *CD320*, for which only one mutation has been described [7]. The cobalamin is then released into the cytosol, an event impaired in people with defects in *LMBRD1* (*cblF* complementation group, MIM #277380) [8]. LMBRD1 protein interacts with ABCD4 protein, which is also involved in the release of *Cbl* into the cytoplasm from the lysosomes (a defect which falls into complementation group *cblJ*) [9]. The most common form of cobalamin defect, complementation group *cblC* (MIM#277400), is caused by mutations affecting *MMACHC* [10]. Patients with mutations in the *Host Cell Factor 1* (*HCFC1*) locus (MIM 309541), located on the X chromosome, have a phenocopy of *cblC* disease [11] since *HCFC1* encodes a transcriptional co-regulator of *MMACHC.* Recently additional defects have been described that affect transcription factors involved in the regulation of Cbl pathway: *ZNF143* [12] and *THAP11* [13]. In addition new variants in *PRDX1* also affect the expression of *MMACHC*, named epi-cblC cases. Thus, these patients result from pathogenic variants in *MMACHC* and one in *PRDX1* which force the antisense transcription of MMACHC and thereby a possible methylation mark [14].

Cellular cobalamin processing occurs via two major pathways - the cytosolic and mitochondrial pathways. The protein thought to be responsible for sorting cobalamin for these pathways is MMADHC (*cblD* complementation group, MIM #611935). This complementation group is the most complex of all since patients present with biochemical heterogeneity ranging from isolated homocystinuria (*cblD* variant 1) or isolated methylmalonic aciduria (*cblD* variant 2) through to methylmalonic aciduria combined with homocystinuria (*cblD*) depending on the nature and location of the mutations present [15].

The cytosolic pathway is involved in the synthesis of MeCbl by MS, encoded by *MTR* (defects give rise to complementation group *cblG*), while the enzyme methionine synthase reductase encoded by *MTRR* is involved in the reactivation of MS (defects give rise to complementation group *cblE*). Both *cblG* (MIM#250940) and *cblE* (MIM#236270) defects, lead to elevated Hcys, hypomethioninaemia, and megaloblastic anaemia [1, 2].

Inactive cob(II)alamin entering the mitochondria is converted into AdoCbl via a reductive adenosylation reaction catalysed by an adenosyltransferase (ATR) encoded at the *cblB* locus (MIM#251110) [16]. MUT is encoded at the *MUT* locus and two different phenotypes have been described: *mut*^*0*^ and *mut*^*−*^ (MIM#251100). Finally *MMAA* is the gene associated with complementation group *cblA* defects (MIM# 607481). It encodes a protein of the same name which may be involved in transferring AdoCbl from ATR to MUT protein as well as maintaining MUT’s functional integrity [5, 17].

Mildly elevated MMA also occurs in patients with mutations affecting *SUCLA2* (MIM#612073) and *SUCLG1* (MIM#245400) which lead to infantile mitochondrial encephalopathic depletion syndrome. These genes encode either the ß-subunit of ADP-forming or the α-subunit of GDP-forming succinyl-CoA synthetases. The accumulated succinyl-CoA inhibits the metabolism of MMACoA to succinyl-CoA, leading to the accumulation of MMA in body fluids. Mutations in *SUCLA2* give rise to typical (though rare) early onset dystonia combined with deafness [18, 19]. An increase in MMA is also seen in patients with mutations in *ACSF3* (MIM# 614265), in whom malonic acid levels may also be elevated (combined malonic and methylmalonic aciduria, CMAMMA) [20]. *ACSF3* encodes a mitochondrial acyl-CoA synthetase considered to have malonyl-CoA and MMACoA synthetase activities.

The diagnosis of an intracellular cobalamin metabolism disorder in a symptomatic individual is based on both clinical suspicion and biochemical analyses [1, 21]. Since expanded newborn screening potentially allows early detection of certain disorders of intracellular cobalamin metabolism, some affected individuals may be diagnosed prior to the onset of clinical symptoms. Diagnosis is based on elevated propionylcarnitine (C3), a high propionylcarnitine to acetylcarnitine ratio (C3/C2), high heptadecanoylcarnitine (C17), and/or reduced methionine concentrations [22]. After symptomatic or asymptomatic detection, a differential diagnosis is needed to help identify the gene that might be involved. Confirmatory testing is based on measuring Hcys and MMA in plasma and/or urine, enzyme analyses, the incorporation of [1-^14^C] propionate and [5-^14^C] methyl-THF into proteins in fibroblasts cultured in basal and hydroxocobalamin-supplemented media, and in some cases by cellular complementation assays [1]. However, biochemical analysis cannot pinpoint a genetic defect, making diagnosis complicated. Fortunately, recent developments in high-throughput sequence capture have made next generation sequencing (NGS) a rapid and accurate means of analysing genetic locus and allele heterogeneous disorders [23–25].

The main purpose of clinical laboratory testing is to support medical decision-making. Clinical genetic testing is generally used to identify or confirm the cause of a disease and, if possible, to provide ideas for personalized treatment. Massive parallel sequencing is the starting point of a complex process for identifying pathogenic variants as well as the description of new functions for a gene product [25]. The aim of the present work was to assess the use of genetic studies as second-tier tests following the detection of cobalamin disorders in biochemical newborn screening. The usefulness of massive parallel sequencing in the identification of the mutated genes responsible for cobalamin and related defects was analyzed, and the structural and functional analysis of the novel variants identified undertaken.

## Methods

### Patients

Patient-derived fibroblasts, blood samples or dried blood spots from 27 patients with elevated MMA and/or Hcys or vitamin B_12_ deficiency were referred to our laboratory for genetic analysis. Another 9 samples from patients with cobalamin defects, previously diagnosed in our laboratory, were included for validation purposes. The diagnosis of these cases was made by biochemical, cellular and genetic studies. Fourteen cases were detected through expanded newborn screening programs; the others were diagnosed after clinical presentation (neonatal or late onset). The study was approved by the ethics committee of the *Universidad Autónoma de Madrid*. The participants or their legal guardians gave their signed, informed consent to be included.

### Genetic analysis

High purity DNA was extracted from whole blood, fibroblasts or dried blood spot samples using the MagNA Pure Compact Kit (Roche Applied Biosciences, Indianapolis, IN) following the manufacturer’s protocol. Two massive parallel sequencing panels were used: targeted customized exome sequencing to capture the exome of 120 genes involved in metabolic disorders (Nextera Nature Capture, Illumina, San Diego, California, USA) (the list of genes included can be sent upon request), and an extended panel (Clinical-Exome Sequencing TruSight™ One Gene Panel, Illumina) that included all the known (in 2013) disease-associated genes described in the OMIM database (Mendeliome panel). For genetic analysis in the validation cohort both panels were used. For genetic analysis in discovery cohort either of the two panels was used, except in cases P24-P27 where both panels were used. After gene capture and alignment with the reference genome, variant callings were made using a virtual panel that included all cobalamin metabolism and related genes. Incidental findings in genes unrelated to the clinical/biochemical phenotypes were ignored. Additional files 1 and 2 describes the coverage of the genes. With both panels, the libraries generated were sequenced using 150 bp paired-end reads employing the Illumina MiSeq or Nextseq500 NGS platforms. SNVs was annotated and subsequently filtered as previously described [26]. Variants were always confirmed by conventional Sanger sequencing employing both patient genomic DNA and that of their parents if available. Pathogenic prediction was assessed by Alamut Visual software. Variants were classified following the ACMG recommendation guidelines [27, 28].

### Assessment of variant pathogenicity

To assess the isolated effect of the recently described mutation c.1084-10A > G in *MUT* [29], the splicing profile was analyzed ex vivo using a minigene assay. Briefly, exon 6 and the corresponding flanking intronic regions from patient P10 were amplified as previously described and cloned into pSPL3 vector [30]. Transcriptional profile analysis was performed as described elsewhere [30].

The oxygen consumption rate (OCR) was measured using the XF24 Extracellular Flux Analyzer (Seahorse Bioscience, Billerica, MA, USA) as previously described [31] in cells transduced with lentiviral particles incorporating wild-type cDNA of *SUCLA2* (NM_003850) or the respective negative control. Modulating compounds such as oligomycin inhibitor of the ATP synthase (OL, 6 μM), carbonyl cyanide 4-(trifluoromethoxy) phenyl-hydrazone a potent uncoupler of oxidative phosphorylation in mitochondria (FCCP, 50 μM), rotenone inhibitor of complex I (RO, 1 μM) and antimycin A inhibitor of complex III (AT, 1 μM) were used to assess the bioenergetic profile and were sequentially added to the cells. The calibration plate for these compounds was prepared according to the manufacturer’s protocol. The data obtained were used to determine basal OCR, the maximum respiratory rate, ATP-linked respiration, mitochondrial reserve capacity, proton leakage and non-mitochondrial respiration. Briefly, for OCR determinations, patient-derived skin fibroblasts (5 × 10^4^ each), were seeded per well in 24-well tissue culture microplates and incubated with 100 μl DMEM supplemented galactose (1 g/L g/l, DMEM-Gal) instead of glucose at 37 °C for 2–5 h. After the incubation period, up to 250 μl of the corresponding medium was added and incubated overnight at 37 °C. On the next day, the medium was removed and the cells washed with phosphate saline buffer (PBS). 700 μl of the corresponding medium DMEM-Gal without bicarbonate were then added and the plates incubated at 37 °C for 1 h.

### Functional prediction

The novel intronic and exonic SNVs identified were analyzed using Alamut Visual software, which includes a number of functional predictors. The two *SUCLA2* variants (p.Gly326Arg, p.Ile312Thr) were mapped to a homology model of the human protein based on the known X-ray structure of SUCLG2 (*S. scrofa*; PDB code: 2fp4) [32]. The percentage sequence identity between these proteins was 54% [33].

## Results

### Genetic analysis

To assess the sensitivity of the proposed assay in the detection of pathogenic mutations, all mapped sequence reads from the nine samples (VC1–9) with previously defined mutations in *TCN2*, *MTR, MTRR, MMACHC, MMADHC, MUT, MMAA* and *MMAB* (Table 1) (11 known and three unknown mutations detected by conventional Sanger sequencing) were inspected blind. Samples for the validation cohort were selected to include different types of *Cbl* deficiency. All the previously known mutations in all samples were detected in their correct heterozygous/homozygous state.

**Table 1:** Genetic and biochemical findings of validation and discovery cases

Reference	Gene	Allele 1 Nucleotide change (protein effect) HGMD number	Allele 2 Nucleotide change (protein effect) HGMD number	Clinical and biochemical findings
VC1	*TCN2* ^*a*^	c.497_498delTC (p.Leu166Profs*7) CD106933	c.497_498delTC (p.Leu166Profs*7) CD106933	2 m pancytopenia, FTT, metabolic acidosis. Improvement after im OHCbl ↑Hcys (pl): 26 μmol/L; ↑C3: 3.4 μmol/L; ↑MMA (ur): 419 mmol/mol creatn Propionate uptake (FB) (nmol/10 h/mg prot): Normal -OHCbl (patient/control): 0.76/0.97 +OHCbl (patient/control): 0.98/0.83
VC2	*MTR* ^*a*^	**c.1348_1349delTCinsGA (p.Ser450Asp)**	c.3008-4A > G (p.?) CS135282	2 m apnea, somnolence, hypotonia, seizures ↑Hcys (pl): 79 μmol/L; MMA (ur) (N)
VC3	*MTRR*	c.1361C > T (p.Ser454Leu) CM032288	**c.1769 + 1G > A (p.Glu560_Arg590del)**	MGA, low serum vit B12; ↑Hcys (pl): 90 μmol/L; N MMA (ur)
VC4	*MMACHC*	c.271dupA (p.Arg91Lysfs*14) CI055013	c.271dupA (p.Arg91Lysfs*14) CI055013	NBS: ↑C3 + C3/C2 in DBS ↑Hcys (pl); ↑MMA (ur) > 1000 mmol/mol creatn
VC5	*MMACHC* ^*a*^	c.271dupA (p.Arg91Lysfs*14) CI055013	c.626dupT (p.Thr210Aspfs*35) CI095519	NBS: ↑C3 in DBS ↑Hcys: 159 μmol/L; C3 (pl): 8.2 μmol/L; ↑MMA (ur): 608 mmol/mol creatn Propionate uptake (FB) (nmol/10 h/mg prot): -OHCbl (patient/control): 0.23/1.90 +OHCbl (patient/control): 2.3/2.34
VC6	*MMADHC* ^*a*^	c.57_64del8 (p.Ser20*) CD082071	c.57_64del8 (p.Ser20*) CD082071	4d hypotonia, metabolic acidosis, hyperammonemia N Hcys (pl); ↑MMA (ur): 5875 mmol/mol creatn; Propionate uptake (FB) (nmol/10 h/mg prot): -OHCbl (patient/control): 0.13/3.31 +OHCbl (patient/control): 2.30/3.37 Mutase (FB):N
VC7	*MUT* ^*a*^	c.312delC (p.Trp105Glyfs*75)	c.1846C > T (p.Arg616Cys) CM050688	12 m metabolic acidosis ↑MMA (ur): 8810 mmol/mol creatn
VC8	*MMAA* ^*a*^	c.64C > T (p.Arg22Ter) CM042745	c.433C > T (p.Arg145Ter) CM042746	5 m metabolic acidosis. Improvement after im OHCbl ↑MMA (ur) Propionate uptake (FB) (nmol/10 h/mg prot): -OHCbl (patient/control): 0.12/1.32 +OHCbl (patient/control): 0.42/2.17 Mutase (FB): N
VC9	*MMAB* ^*a*^	c.548A > T (p.His183Leu) CM154654	c.570_572dupCCG (p.Arg191dup) CI154655	NBS: ↑C3 in DBS ↑C3 (pl): 7.7 μmol/L; ↑MMA (ur): 830 mmol/mol creatn Propionate uptake (FB) (nmol/10 h/mg prot): -OHCbl (patient/control): 0.63/1.90 +OHCbl (patient/control): 1.19/2.34 Mutase (FB): N
P1	*TCN1*	c.901G > T^b^ (p.Asp301Tyr) CM099555	c.901G > T^b^ (p.Asp301Tyr) CM099555	Adult with neurological symptoms and low serum vit B_12_. Improvement after im OHCbl ↑N Hcys (pl): 17 μmol/L
P2	*TCN1*	c.901G > T^b^ (p.Asp301Tyr) CM099555	c.901G > T^b^ (p.Asp301Tyr) CM099555	Adult with slight anemia and low serum vit B12. Improvement after im OHCbl
P3	*GIF* ^*a*^	**c.389C > G (p.Ser130Ter)**	**c.389C > G (p.Ser130Ter)**	7y, anemia since 15 m; low serum vit B12. Improvement after im OHCbl ↑Hcys (pl): 49 μmol/L
P4	*AMN* ^*a*^	c.514-34G > A (p.Thr172fs) CS127867	**c.1046C > A (p.Ser349Ter)**	2y, megaloblastic anemia with low serum vit B12. Improvement after im OHCbl ↑Hcys (pl): 39.9 μmol/L
P5	*MMAA*	c.593_596delCTGA (p.Thr198Serfs*6) CD043681	c.593_596delCTGA (p.Thr198Serfs*6) CD043681	↑MMA (ur)
P6	*MUT* ^*a*^	**c.904G > C (p.Ala302Pro)**	**c.904G > C (p.Ala302Pro)**	NBS: ↑C3 + C3/C2 in DBS ↑MMA (ur): > 1000 mol/mol creatn Propionate uptake (FB) (nmol/10 h/mg prot) - > (mut0) -OHCbl (patient/control): 0.18/1.16 +OHCbl (patient/control): 0.11/1.02
P7	*MUT* ^*a*^	c.671_678dupAATTTATG (p.Val227Asnfs*16) CI050942	c.607G > A (p.Gly203Arg) CM002067	Neonatal presentation ↑MMA (ur)
P8	*MUT*	c.313 T > C (p.Trp105Arg) CM900166	c.2150G > T (p.Gly717Val) CM920488	7 m metabolic acidosis, lethargy ↑MMA (ur)
P9	*MUT* ^*a*^	**c.2026G > A (p.Ala676Thr)**	**c.2026G > A (p.Ala676Thr)**	NBS: ↑C3/C2 in DBS ↑C3 (pl): 2.4 μmol/L; ↑MMA (ur): 772 mmol/mol creatn Propionate uptake (FB) (nmol/10 h/mg prot) - > (mut-) -OHCbl (patient/control): 0.25/1.16 +OHCbl (patient/control): 1.16/1.02
P10	*MUT* ^*a*^	c.243dupA (p.Pro82Thrfs*2)	c.1084-10A > G (p.?) CS128372	↑MMA (ur)
P11	*MUT* ^*a*^ TCN1^*a*^	c.454C > T (p.Arg152Ter) CM050671 c.901G > T^b^ (p.Asp301Tyr) CM099555	?	NBS: ↑C3 in DBS Low serum vit B12; ↑N MMA (ur): 17–58 mmol/mol creatn
P12	*MUT*	c.671_678dupAATTTATG (p.Val227Asnfs*16) CI050942	c.671_678dupAATTTATG (p.Val227Asnfs*16) CI050942	Neonatal presentation and fatal outcome ↑MMA (ur)
P13	*MUT* ^*a*^	c.1420C > T (p. Arg474Ter) CM050685	**c.2026G > A (p.Ala676Thr)**	NBS: ↑C3/C2 in DBS ↑C3 (pl): 3.4 μmol/L; ↑MMA (ur): 276 mmol/mol creatn Propionate uptake (FB) (nmol/10 h/mg prot) - > (mut-) -OHCbl (patient/control): 0.28/1.33 +OHCbl (patient/control): 1.13/1.57
P14	*MMAB* ^*a*^	**c.220G > T (p.Glu74Ter)**	c.548A > T (p.His183Leu) CM154654	NBS: ↑C3/C2 in DBS ↑C3 (pl): 3.64 μmol/L; ↑MMA (ur): 68 mmol/mol creatn
P15	*CD320* ^*a*^	c.262_264delGAG (p.Glu88del) CD104789	**c.142 + 5G > A (p.?)**	NBS: ↑C3 + C3/C2 in DBS ↑Hcys (pl): 18.3 μmol/L; N C3 + C4DC (pl); ↑MMA (ur): 25–170 mmol/mol creatn
P16	*MMACHC* ^*a*^	c.347 T > C (p.Leu116Pro) CM060067	c.347 T > C (p.Leu116Pro) CM060067	NBS: ↑C3 + C3/C2 in DBS ↑MMA (ur): 2000 mmol/mol creatn
P17	*MMACHC* ^*a*^	c.271dupA (p.Arg91Lysfs*14) CI055013	c.271dupA (p.Arg91Lysfs*14) CI055013	4d: hypotonia, weight loss, vomiting, ↑lac ↑Hcys (pl): 214 μmol/L; ↑C3: 6.5 μmol/L + ↑C4DC (pl): 0.25 μmol/L ↑MMA (ur): 1197 mmol/mol creatn
P18	*MMACHC*	c.271dupA (p.Arg91Lysfs*14) CI055013	c.271dupA (p.Arg91Lysfs*14) CI055013	↑Hcys (pl); ↑MMA (ur)
P19	*MMADHC* ^*a*^	c.748C > T (p.Arg250Ter) CM081188	c.748C > T (p.Arg250Ter) CM081188	NBS: ↑C3 + C3/C2 in DBS ↑Hcys (pl): 51 μmol/L; ↑C3 (pl): 4.8 μmol/L; MMA (ur): 290 mmol/mol creatn
P20	*MMADHC* ^*a*^	c.748C > T (p.Arg250Ter) CM081188	c.748C > T (p.Arg250Ter) CM081188	NBS: ↑C3 + C3/C2 in DBS ↑Hcys (pl): 59 μmol/L; ↑C3 (pl): 8.5 μmol/L; MMA (ur): 1175 mmol/mol creatn
P21	*MTRR*	c.1361C > T (p.Ser454Leu) CM032288	c.1361C > T (p. Ser454Leu) CM032288	17 m; anemia, psychomotor delay ↑Hcys (pl): 76 μmol/L
P22	*MTRR*	c.1361C > T (p.Ser454Leu) CM032288	c.1678_1681delGAGA (p.Glu560Asnfs*42) CD159487	10 m seizures, megaloblastic anemia ↑Hcys (pl): 89 μmol/L
P23	*SUCLA2* ^*a*^	**c.976G > C (p.Gly326Arg)**	**c.935 T > C (p.Ile312Thr)**	Hypoglycemia at birth, slight PMD during first years Asymptomatic at 11y ↑-N C3(pl): 1–2.2 μmol/L + C4DC ↑N Lac, 3-OHProp, MMA 27 mmol/mol creatn, MC (ur) Normal carboxylases activities (FB) Propionate uptake (FB) (nmol/10 h/mg prot) - > Normal -OHCbl (patient/control): 0.71/0.75 +OHCbl (patient/control): 0.79/1.13
P24	*ACSF3* ^*a*^	**c.424C > T (p.Gln142Ter)**	**c.1446_1447delCA (p.Tyr482Ter)**	NBS: ↑MMA (ur) since newborn: 512 mmol/mol creatn Asymptomatic at 3y
P25	*ACSF3* ^*a*^	c.1075G > A (p.Glu359Lys) CM116777	c.1075G > A (p.Glu359Lys) CM116777	5y PMD, bilateral sensorineural hearing loss AC (pl): N; ↑MMA (ur): 360 mmol/mol creatn
P26	*ACSF3* ^*a*^	c.1075G > A (p.Glu359Lys) CM116777	c.1672C > T (p.Arg558Trp) CM116840	Seizures since first months of age At 12y seizures + dystonia AC (pl): N; ↑MMA (ur): 124–560 mmol/mol creatn Normal propionate uptake ± OHCbl (FB)
P27	*ACSF3*	**c.820C > T (p.Gln274Ter)**	**c.820C > T (p.Gln274Ter)**	NBS: ↑MMA (ur) since newborn. Asymptomatic at 7 m

*VC* validation case, *C3* propionylcarnitine, *C2* acetylcarnitine, *C4DC* methylmalonylcarnitine, *DBS* dried blood spots, *FB* fibroblasts, *Hcys* homocysteine, *im* intramuscular, *MMA* methylmalonic acid, *OHCbl* hydroxocobalamin, *ur* urine, *pl* plasma, *Lac* Lactate, *MC* methylcitrate, *NBS* newborn screening, *PMD* psychomotor delay

^a^Mutations confirmed in parents; ^b^ Variant with uncertain clinical significance; new mutations in bold

Mutations named according to GenBank accession number included in Additional file 3: Table S1

The discovery cohort consisted of samples received for cobalamin genetic diagnosis, for which no mutations were previously known. Variant changes were identified in the cobalamin transport genes i.e., *TCN1*, *GIF* and *AMN*, in genes causing isolated MMA i.e., *MMAA, MUT* and *MMAB*, in genes causing MMA&HC i.e., *CD320*, *MMACHC*, *MMADHC*, in genes causing HC i.e., *MTRR*, and in two MMA metabolism-related genes *SUCLA2* and *ACSF3* (Table 1). Some variants in *MMACHC, MMADHC, MUT, MTRR* and *ACSF3* were detected in several samples. In some cases DNA from blood spots was used in parallel; the coverage compared to whole blood was similar.

Overall, 33 variations were identified in 12 different genes. All the new exonic non-synonymous SNVs (nsSNVs) were predicted to be pathogenic or likely pathogenic (Additional files 1 and 3).

Among 27 samples with biallelic nucleotide changes, 21 showed at least one described (HGMD® professional release professional release 2018.1) or one new LoF mutation. Three samples (P6, P9 and P23) presented two new variants in *MUT* or *SUCLA2.* A previously described functional SNP in *TCN1* was detected in two samples (P1 and P2) [4]. Although this disease-associated polymorphism was not detected in the in-house control samples analysed, this variation is present close to 9% in all data base consulted (ExAc, gnomAD and Spanish data) with presence of homozygous cases. Therefore this change alone in *TCN1* is unlikely to be responsible for the clinical picture in P1 and P2.

P11 (identified by newborn screening) presented the previously described nonsense mutation p.Arg152Ter in *MUT,* plus the disease-associated polymorphism in *TCN1* described above. Both changes were present in the paternal sample. No other exonic or described intronic mutation were detected. Mean coverage allowed to rule out a large genomic deletion. It was not possible to rule out the presence of deep intronic mutations.

Four patients (P24, P25, P26 and P27) referred for analysis of genes related to isolated MMA in fact had mutations in *ACSF3*. After the genetic diagnosis, a slight increase in urinary malonic acid was confirmed by GC-MS in three of them.

### Functional analysis of the *MUT* splicing mutation c.1084-10A > G

**Fig. 1:**
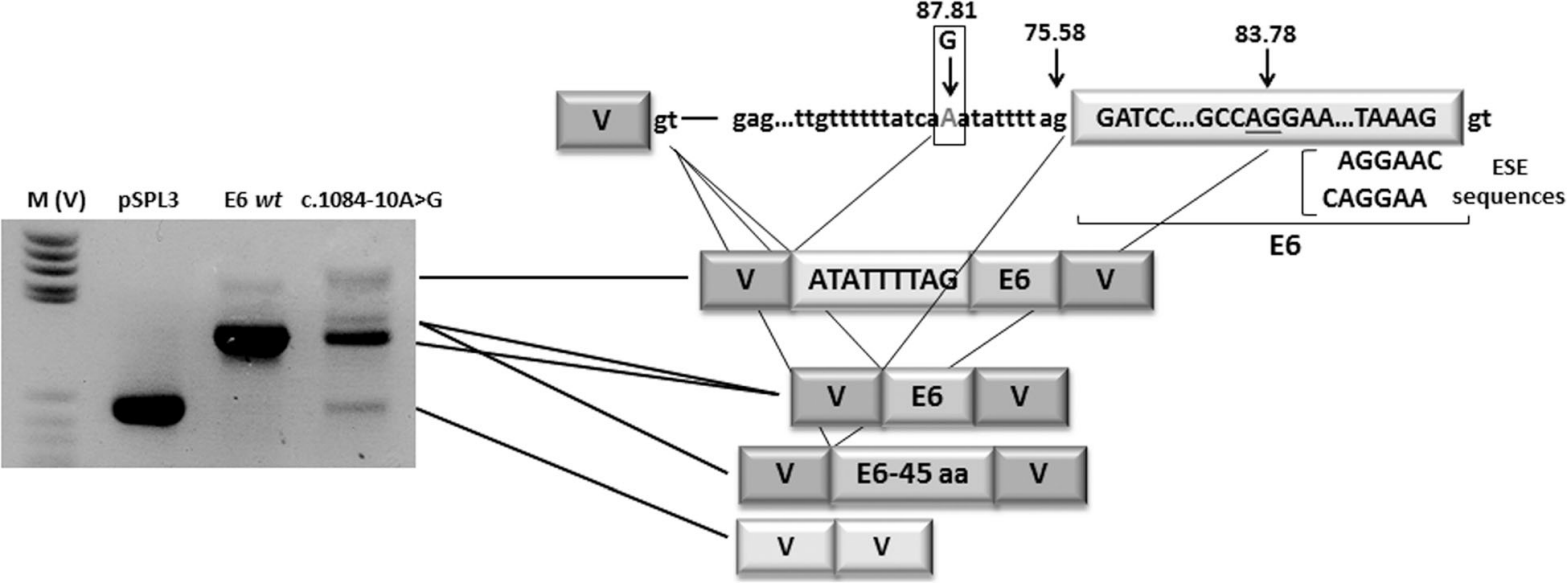
Functional analysis of c.1084-10G > A identified in the *MUT* gene. Splicing pattern observed in the minigene construct bearing the empty vector (pSPL3), normal minigene (c.1084-10G), and mutant vector c.1084-10G. The figure shows the splicing scores obtained using the Human Splicing Finder (www.umd.be/HSF3/). The ESE sequences were obtained using RESCUE-ESE software (www.genes.mit.edu/burgelab/rescue-ese)

The effect of mutation c.1084-10A > G identified in P10 on the splicing process was addressed using an ex vivo minigene system. The results indicate the insertion of a 9 nt sequence upstream of the normal 3′ splice site, as recently described by transcriptional profile analysis in patient-derived fibroblasts [29]. A shorter transcript was also observed, resulting from the deletion of 135 nt from exon 6. This was detected as a heteroduplex along with the normal transcript. This deletion was generated by the activation of an internal exonic cryptic 3’splice with a higher splicing score than normal, plus two exonic splicing enhancer sequences (Fig. 1).This deletion would likely translate into the elimination of 45 amino acid residues from the protein.

### Functional analysis of *SUCLA2* defects in patient-derived fibroblasts

**Fig. 2:**
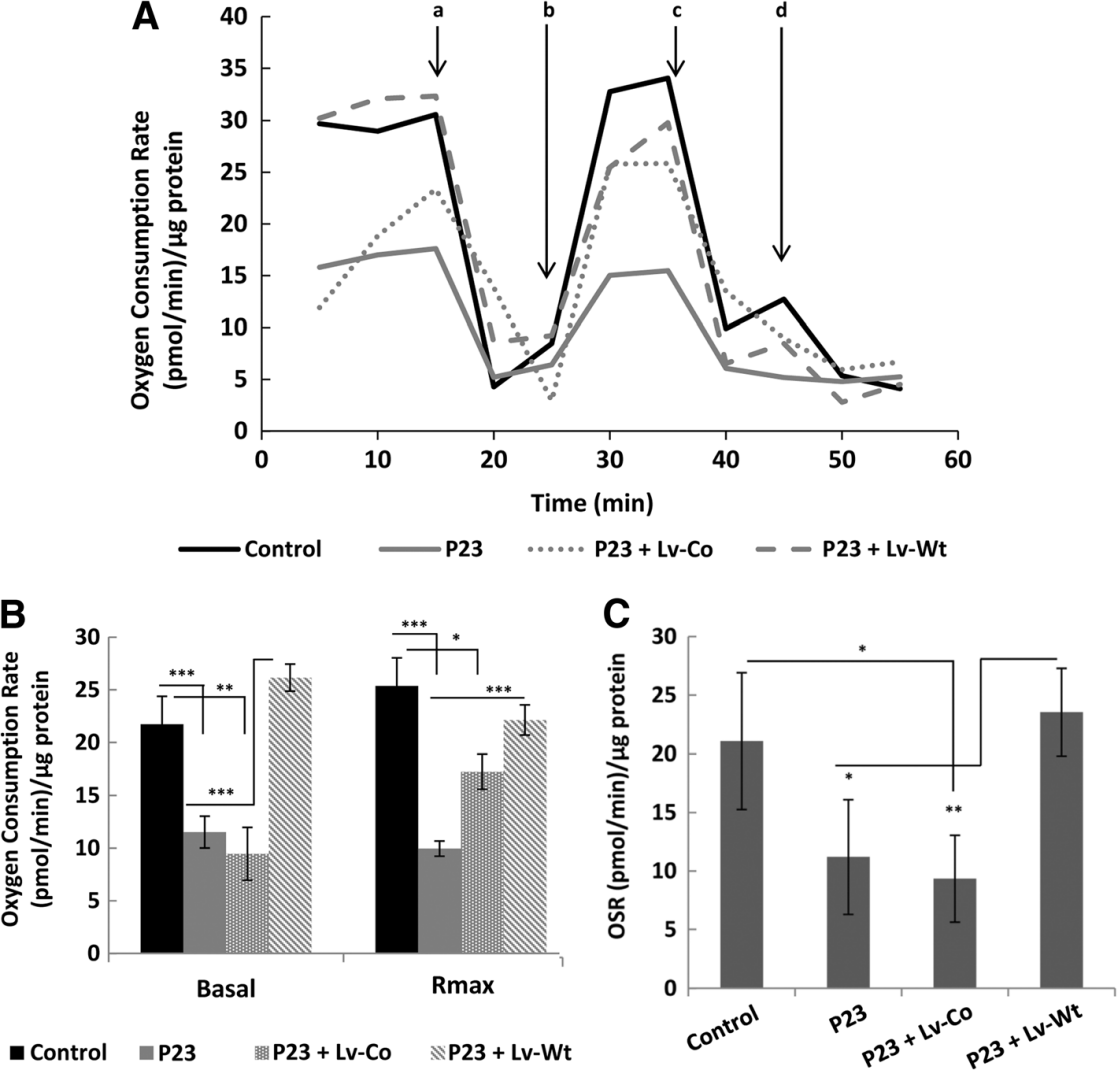
Functional rescue of mitochondrial dysfunction. Bioenergetic profiles of *SUCLA2* in patient P23 fibroblasts transduced with a lentiviral plasmid bearing wild-type *SUCLA2* cDNA (Lv Wt) or a null construct (Lv Co), and control-derived fibroblasts. Experiments were performed using a Seahorse XF device. **A** Oxygen consumption rates of control and patient fibroblasts were measured in DMEM with galactose (1 g/L) instead of glucose, and upon the subsequent addition of **a** 6 μM oligomycin, **b** 50 μM FCCP, **c** 1 μM rotenone or, **d** 1 μM antimycin. **B** Basal and maximum respiration (Rmax) were calculated for each situation. **C** Oligomycin-sensitive respiration (OSR) was calculated as the difference between the basal respiration and the oxygen consumption rate measured as described in (**C**), after the addition of 6 μM oligomycin. The results reflect the mean of three biological repetitions. Control values are the mean for two different control cell lines. (*p < 0.05; **p < 0.01; ***p < 0.001 [Student t test])

To assess the bioenergetic profile of patient P23, and to determine whether mitochondrial function could be restored by the transfection of patient-derived fibroblasts with a lentiviral construct bearing wild-type *SUCLA2* cDNA, the oxygen consumption rate (OCR) of control and patient derived-fibroblasts (transduced or not with the lentiviral construct) was analyzed. In order to force the functioning of the electron transport chain, this experiment was performed in a medium supplemented with galactose. Fig. 2a shows that P23-derived fibroblasts had a diminished bioenergetic profile compared to control cells. Basal respiration was significantly reduced (Fig. 2b). The addition of FCCP to the medium resulted in increased electron flow through the mitochondrial respiratory chain and allowed the maximum respiration rate to be calculated. Figure 2b shows this variable to also be significantly reduced in P23 fibroblasts. Oligomycin-sensitive respiration (OSR), which represents the ATP-linked oxygen consumption (Fig. 2c), was also reduced in P23 cells, indicating reduced oxidative phosphorylation.

The transduction of patient dermal fibroblasts with an empty construct (Lv-Co) did not influence the bioenergetic profile, except for an increase in the maximum respiration rate which was still below control levels. Transduction with the lentiviral construct bearing wild-type *SUCLA2* cDNA, however, was able to restore the bioenergetic profile (Fig. 2a). Both basal and maximal respiration rates were restored to control levels (Fig. 2b). As shown in Fig. 2c, oligomycin-sensitive respiration was also significantly increased, suggesting the restoration of oxidative phosphorylation. These results indicate that the bioenergetic deficit observed in the cells from P23 could be caused by defects in *SUCLA2*.

### In silico estimates of the functional impact of missense variants

Structural analysis of the *SUCLA2* variants was performed for prediction of protein dysfunction at the molecular level.

**Fig. 3:**
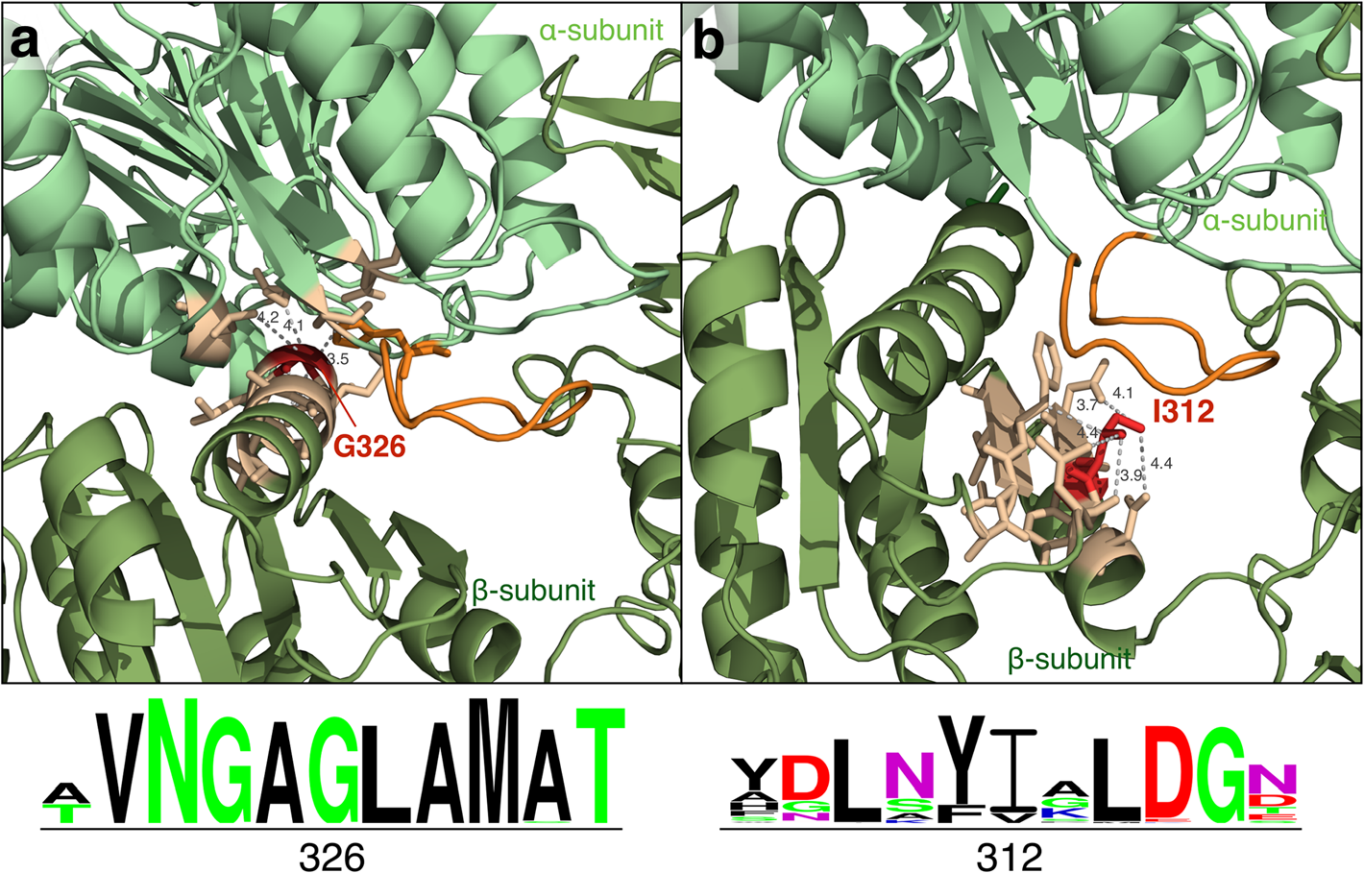
Structure and conservation analysis of *SUCLA2* variants (p.G326R, p.I312T). For each variant, p.G326R (**a**) or p.I312T (**b**), the location of the wild-type amino acid (red) and its network of interactions (light orange) are shown. Close residue-residue contacts are drawn with dashed lines. The “phosphate shuttle” loop is marked in dark orange [44]. The conservation pattern for each variant is represented below the structural models. The size of the letters reflects the degree of conservation

The functional and in silico analyses of the two *SUCLA2* variants also suggested them to be damaging. Only AlignGVGD gave a nearly neutral score for p.Ile312Thr. It should be noted that the pathogenicity score for this variant was closer to the threshold between neutral and damaging replacements, suggesting a milder effect (Additional file 1). This observation agrees with the results of the structural analysis. The wild type residues Gly326 and Ile312 were identified in a homology model of *SUCLA2*, obtained using pig *SUCLG2*. Visual analysis of the structures (Fig. 3) showed that both loci fell near the protein’s “phosphate shuttle” loop [18], indicating that the replacement of the wild-type residue might interfere with the functional role of this loop. This was particularly noticeable for Gly326, which lies in close contact with residue His299 of the loop, and occurs at a packed location in the structure. The replacement of glycine by the bulkier arginine would undoubtedly create some steric clashes. Gly326 was found to be highly conserved in multiple sequence alignments of the *SUCLA2* family, supporting the idea that it may have a functional role. Finally, Ile312 was involved in a dense network of residue interactions, although no close contact (<5Angs) with the residues from the functional “phosphate shuttle” loop was observed. This indicates, once again, that replacement of Ile312 may have a milder impact on function. This agrees with the less strict conservation pattern seen in multiple sequence alignments. In summary, agreement was seen between in silico predictions and structural analyses for both variants, indicating that the observed replacements are pathogenic, although p.Gly326Arg probably causes more severe problems than p.Ile312Thr.

## Discussion

The gold standard for coming to genetic diagnoses of cobalamin defects has for some time been gene-by-gene Sanger sequencing of individual DNA fragments. Enzymatic and cellular methods are employed before such sequencing to help in the selection of the gene defects to be sought, but this is time-consuming and laborious [1]. Further, no biochemical methods have been available to test for cobalamin absorption and transport defects, and while plasma B_12_ concentrations are tentatively used to help in the selection of genes to be sequenced, this has probably led to the under-recognition of cases [34]. This costly, stepwise, and time-consuming methodology is gradually being replaced by NGS technologies, which offer higher throughput and scalability, cost less per sequenced nucleotide and have shorter turnaround times [35]. The present work reports the NGS analysis of a cohort of patients suspected of having cobalamin defects (plus the corresponding structural/functional analyses for some of the defects involved) and shows it to be quick and reliable.

A 100% correct diagnostic rate was returned with the validation cohort, with exonic and intronic nucleotide changes and small deletions (c.271dupA in *MMACHC* or c.57_64del8 in *MMADHC*) all successfully detected. With the clinical exome massive parallel sequencing system used, the depth and breadth of coverage of the cobalamin metabolism-related genes was > 30× and 99% respectively. The sensitivity and specificity of the method appeared to be very good and the results reproducible (several mutations were detected in more than one unrelated mutant allele i.e., c.271dupA in *MMACHC*, c.671_678dupAATTTATG in *MUT*, c.748C > T in *MMADHC* etc**.**). Thus, even though the use of smaller, specific panels is recommended [36] so that secondary incidental findings are avoided, the present work shows clinical exome sequencing to be successful. It could be used in sequencing analyses of single patients as well groups of patients whose members have other, unrelated disorders*.*

The LoF mutations detected in *GIF* and *AMN* were found to affect the absorption of cobalamin. *GIF* and *AMN*, in addition to *CUBN* and *TCN1*, are not usually captured in the available panels offered by genetic companies but the results reported in the present work have demonstrated they should be analyzed [19, 34].

The potential missense changes p.Ala302Pro and p.Ala676Thr, identified in *MUT*, were predicted to be damaging by several bioinformatic algorithms. The present detection of the associated mutations in combination with functional cellular studies provided a rational basis for tailored treatment. Indeed, the cellular uptake of ^14^C-propionate revealed that p.Ala302Pro is a *mut*^*0*^ mutation, while p.Ala676Thr located in the C-terminal of the cobalamin binding domain is likely a *mut*^−^ mutation. Based on these results, patient P6 might not be responsive to the pharmacological administration of the vitamin, while P9 would likely respond. Case P11 inherited a described LoF mutation in *MUT* and the functional polymorphism p.Asp301Tyr in *TCN1*, probably an incidental finding of no clinical significance. This asymptomatic individual was detected in newborn screening. No other mutations were detected in the coding region of *MUT*, not even in the deep intronic sequence reported to bear an intronic pathogenic mutation [30, 37]. Although massive parallel sequencing exome analysis cannot rule out the presence of other nucleotide changes in promoter or intronic sequences, both the absence of clinical symptoms and the slight abnormal levels of urinary MMA in case P11 suggested he/she was a carrier of the disease.

Variations in genes that do not encode cobalamin metabolism-related proteins (*ASCF3* and *SUCLA2*) were also detected in the present work. Both were associated with slightly increased urinary MMA. The two new variants of *SUCLA2* (p.Gly326Arg and p.Ile312Thr) detected are probably damaging changes; both were predicted to be so in bioinformatic and structural analyses. Neither was found to be present in database control populations nor in the in-house control samples. Structural analysis suggested that the replacement of Ile312 may have a mild functional impact, which agrees with the less strict conservation pattern seen in multiple sequence alignments. Importantly, the mitochondrial dysfunction in deficient fibroblasts from P24 was rescued via the stable expression of the SUCLA2 protein through lentiviral transduction. This suggests that *SUCLA2* is the gene likely affected in this patient, although his/her symptoms were atypical [18, 38]. With respect to *ASCF3*, individuals P24, P25, P26 and P27 were diagnosed as having isolated MMA since the urinary malonic acid that identifies CMAMMA can be easily missed [39]. Given the difficulty in diagnosing CMAMMA, several of the present patients were originally diagnosed during genetic analyses, e.g., exome sequencing [40]. Even though a new method for the rapid metabolic diagnosis of CMAMMA has recently been reported [39], diagnoses by genetic analysis would be improved by second tier genetic testing after newborn screening. In some cases *ACSF3* defects only cause biochemical conditions such as short-chain acyl-CoA dehydrogenase deficiency [41] or 3-methylcrotonylglycinuria [42], and symptoms appear only during viral infections etc. [39, 43]. In other cases, as reported in the present work, patients can show neurological damage [19]. Prompt diagnosis would avoid unnecessary treatment with vitamin B_12_.

The use of massive parallel sequencing in a new era of newborn screening would avoid the time-consuming biochemical and enzymatic methods presently used to help identify which genes might be causing isolated MMA, MMA&HC, HC or CMAMMA. The rapid return of an accurate genetic diagnosis would allow the prescription of an appropriate treatment just a few days after birth. This report illustrates the clinical usefulness of massive parallel sequencing in the diagnosis of cobalamin and related defects. This powerful technology could improve the detection of these under-recognized or rare vitamin B_12_ defect conditions.

## Conclusions

This work provides evidence supporting the use of massive parallel sequencing as second tier test for confirming cobalamin defects following their detection in newborn screening. Biochemical and cellular analyses and additionally comprehensive phenotyping are needed for an accurate classification of variants for making fully useful clinical reports.

## Additional files

Additional file 1: New and VUS missense mutations in cobalamin genes identified in the discovery cohort. (DOCX 24 kb)

Additional file 2: Functional prediction of new splicing changes identified in cobalamin gene defects. (DOCX 20 kb)

Additional file 3: Horizontal and vertical coverages of cobalamin and related genes. (DOCX 24 kb)

## Abbreviations

AdoCbl: Adenosylcobalamin
ATR: Adenosyltransferase
CMAMMA: Combined malonic and methylmalonic aciduria
FCCP: Carbonyl cyanide 4-(trifluoromethoxy) phenyl-hydrazone
Hcys: Homocysteine
MeCbl: Methylcobalamin
MMA: Methylmalonic acid
MMA&HC: Methylmalonic aciduria with homocystinuria
MMACoA: L-methylmalonyl-CoA
MS: Methionine synthase
MUT: Methylmalonyl-CoA mutase
NGS: Next generation sequencing
OCR: Oxygen consumption rate
TCblR: Transcobalamin receptor
