# Supplementary material for: Improving the diagnosis of cobalamin and related defects by genomic analysis, plus functional and structural assessment of novel variants
Source: Orphanet J Rare Dis. 2018 Jul 24;13:125. doi: 10.1186/s13023-018-0862-y (PMC6057060; doi:10.1186/s13023-018-0862-y)
Supplement: Supplementary file 1 — New and VUS missense mutations in cobalamin genes identified in the discovery cohort. (DOCX 24 kb) [file 13023_2018_862_MOESM1_ESM.docx]

**S2 Table**: New and VUS missense mutations in cobalamin genes identified in the discovery cohort

| **Gene**  **Transcript** | **Mutation** |  | **Prediction (AlamutVisual®)** | | | | | | | **Allele frequency** | | |  |
| --- | --- | --- | --- | --- | --- | --- | --- | --- | --- | --- | --- | --- | --- |
|  |  | **Exon** | **PhyloP^d^** | **GVGD** | **SIFT** | **Polyphen2** | **Mutation taster ^c b^** | **Provean** | **Mutation**  **assessor** | **MAF ^e^** | **EVS ^f^** | **ExAc ^g^** | **Classification** |
| *MUT*  NM_000255.3 | c.904G>C  p.Ala302Pro  Chr6g.49423800 | 4 | 5.4  [-14.1;6.4] | C0  (GV: 208.40 - GD: 2.22) | Deleterious (score: 0) | probably damaging  (score: 1) | disease causing | Deleterious -4.994 | high  score  4.54 | - | - | - | Likely pathogenic (IV) |
| *MUT*  NM_000255.3  rs147715336 | c.2026G>A  p.Ala676Thr  Chr6g.49403267 | 12 | 5.53  [-14.1;6.4] | C55  (GV: 0.00 - GD: 58.02) | Deleterious (score: 0) | probably damaging  (score: 1) | disease causing | Deleterious -3.812 | high  score  3.535 | 0 | 0% | 0.0083% | Likely pathogenic (IV) |
| *SUCLA2*  NM_003850.2 | c.976G>C  p.Gly326Arg  Chr13g.48528406 | 8 | 3.84  [-14.1;6.4] | C65  (GV: 0.00 - GD: 125.13) | Deleterious (score: 0) | probably damaging  (score: 1) | disease causing | Deleterious -7.507 | high  score  4.685 | - | - | - | Likely pathogenic (IV) |
| *SUCLA2*  NM_003850.2 | c.935T>C  p.Ile312Thr  Chr13g.48528560 | 7 | 4.97  [-14.1;6.4] | C25  (GV: 28.68 - GD: 69.84) | Deleterious (score: 0.01) | possibly damaging  (score: 0.801) | disease causing | Deleterious -4.522 | medium  score  2.45 | - |  |  | Likely pathogenic (IV) |

ND. no data. Genomic coordinates are given in hg19/GRCh37. Nomenclature of the mutations was done following the recommendations of Human Genome Variation Society (HGVS) and checked using Mutalyzer (<https://mutalyzer.nl>).
