# Supplementary material for: Improving the diagnosis of cobalamin and related defects by genomic analysis, plus functional and structural assessment of novel variants
Source: Orphanet J Rare Dis. 2018 Jul 24;13:125. doi: 10.1186/s13023-018-0862-y (PMC6057060; doi:10.1186/s13023-018-0862-y)
Supplement: Supplementary file 2 — Functional prediction of new splicing changes identified in cobalamin gene defects. (DOCX 20 kb) [file 13023_2018_862_MOESM2_ESM.docx]

**S3 Table** : Functional prediction of new splicing changes identified in cobalamin gene defects

| **Gene**  **(Transcript)** | **Mutation** | **Prediction (AlamutVisual®)** | | | | |
| --- | --- | --- | --- | --- | --- | --- |
|  |  | **SSF [0-100]** | **MaxEnt [0-16]** | **NNSPLICE [0-1]** | **GeneSplicer [0-15]** | **HSF [0-100]** |
| *CD320*  NM_016579.3 | c.142+5G>A  p.? | 95.64 ⇒ 83.49 (-12.7%) | 10.67 ⇒ 6.60 (-38.2%) | 0.99 ⇒ 0.71 (-28.9%) | 20.93 ⇒ 10.29 (-50.8%) | 98.84 ⇒ 86.67 (-12.3%) |
| *MUT*  NM_000255.3  CS128372 | c.1084-10A>G  p.? | 72.17 ⇒ — | 10.04 ⇒ 3.06 (-69.5%) | 0.42 ⇒ 0.55 (+30.3%) | 3.03 ⇒ — | 75.47 ⇒ 75.58 (+0.2%) |

^a^ <http://sift.jcvi.org/>. ^b^ <http://genetics.bwh.harvard.edu/pph2/>. ^c^ <http://www.mutationtaster.org/>. ^d^ http://provean.jcvi.org/

Genomic coordinates are given in hg19/GRCh37. Nomenclature of the mutations was done following the recommendations of Human Genome Variation Society (HGVS) and checked using Mutalyzer (<https://mutalyzer.nl>).
