# Supplementary material for: Improving the diagnosis of cobalamin and related defects by genomic analysis, plus functional and structural assessment of novel variants
Source: Orphanet J Rare Dis. 2018 Jul 24;13:125. doi: 10.1186/s13023-018-0862-y (PMC6057060; doi:10.1186/s13023-018-0862-y)
Supplement: Supplementary file 3 — Horizontal and vertical coverages of cobalamin and related genes. (DOCX 24 kb) [file 13023_2018_862_MOESM3_ESM.docx]

| S1 Table: Horizontal and vertical coverages of cobalamin and related genes | | | | | | | | | | | | |  | | | | | | |
| --- | --- | --- | --- | --- | --- | --- | --- | --- | --- | --- | --- | --- | --- | --- | --- | --- | --- | --- | --- |
|  |  |  | |  | |  | | **Trusight^TM^ One** | | | | | **Nextera Rapid Capture** | | | | | | |
|  |  | | | | | | Horizontal coverage | | | Vertical coverage | | | Horizontal coverage | | | Vertical coverage | | |  |
| Gen | Disease | OMIM | Coordinates (hg19) | | REFSEQ  NM | | Target size (bp) | | % bp  not cov | Mean cov | % bp  cov < 20x | % bp  cov ≥ 30x | Target size (bp) | % bp not cov | | Mean cov | % bp  cov < 20x | % bps  cov ≥ 30x |  |
| *MUT* | ***MMA*** | *609058* | chr6: 49,398,073-49,430,904 | | [000255.3](http://www.ncbi.nlm.nih.gov/entrez/viewer.fcgi?val=NM_000255.3) | | 2493 | | 0 | 92.9 | 1.5 | 98.5 | 3886 | 0 | | 158.2 | 0 | 100 |  |
| *MMAA* | ***MMA*** | *607481* | chr4: 146,539,415-146,581,187 | | [172250.2](http://www.ncbi.nlm.nih.gov/entrez/viewer.fcgi?val=NM_172250.2) | | 1377 | | 0 | 126.3 | 1.2 | 98.5 | 5943 | 0.3 | | 186.7 | 0.3 | 99.5 |  |
| *MMAB* | ***MMA*** | *607568* | chr12: 109,991,542-110,011,679 | | [052845.3](http://www.ncbi.nlm.nih.gov/entrez/viewer.fcgi?val=NM_052845.3) | | 4425 | | 0 | 100.3 | 0.1 | 99.9 | 4225 | 0 | | 276.5 | 0 | 100 |  |
| *MMADHC* | ***MMA*** | *611935* | chr2: 150,426,148-150,444,330 | | [015702.2](http://www.ncbi.nlm.nih.gov/entrez/viewer.fcgi?val=NM_015702.2) | | 1031 | | 0 | 86.6 | 0.5 | 99.3 | 1437 | 0 | | 111.2 | 0.1 | 99.4 |  |
| *MCEE* | ***MMA*** | *608419* | chr2: 71,336,814-71,357,369 | | [032601.3](http://www.ncbi.nlm.nih.gov/entrez/viewer.fcgi?val=NM_032601.3) | | 591 | | 0 | 71.3 | 0.2 | 98.9 | 870 | 0 | | 175.9 | 0.4 | 99.6 |  |
| *SUCLG1* | ***MMA*** | *611224* | chr2: 84,650,647-84,687,169 | | [003849.3](http://www.ncbi.nlm.nih.gov/entrez/viewer.fcgi?val=NM_003849.3) | | 1221 | | 0 | 88.2 | 0.1 | 99.7 | 1457 | 0 | | 169.2 | 0 | 100 |  |
| *SUCLA2* | ***MMA*** | *603921* | chr13: 48,510,622-48,612,125 | | [003850.2](http://www.ncbi.nlm.nih.gov/entrez/viewer.fcgi?val=NM_003850.2) | | 1612 | | 0 | 100.4 | 0 | 99.6 | 2164 | 0 | | 108.1 | 0.1 | 99.7 |  |
| *MMACHC* | ***MMAHC*** | *609831* | chr1: 45,965,725-45,976,739 | | [015506.2](http://www.ncbi.nlm.nih.gov/entrez/viewer.fcgi?val=NM_015506.2) | | 929 | | 0 | 108.7 | 2.1 | 97.9 | 2850 | 0 | | 182.3 | 0 | 100 |  |
| *MMADHC* | ***MMAHC*** | *611935* | chr2: 150,426,148-150,444,330 | | [015702.2](http://www.ncbi.nlm.nih.gov/entrez/viewer.fcgi?val=NM_015702.2) | | 1031 | | 0 | 86.6 | 0.5 | 99.3 | 1437 | 0 | | 111.2 | 0.1 | 99.4 |  |
| *LMBRD1* | ***MMAHC*** | *612625* | chr6: 70,385,694-70,507,003 | | [018368.3](http://www.ncbi.nlm.nih.gov/entrez/viewer.fcgi?val=NM_018368.3) | | 1943 | | 0 | 68.4 | 0.2 | 99.6 | 2308 | 0.1 | | 95.1 | 3.5 | 96.5 |  |
| *TCN2* | ***MMAHC*** | *613441* | chr22: 31,002,825-31,023,265 | | [000355.3](http://www.ncbi.nlm.nih.gov/entrez/viewer.fcgi?val=NM_000355.3) | | 1464 | | 0 | 78.1 | 0.3 | 99.2 | 2161 | 0 | | 341.5 | 0 | 100 |  |
| *GIF* | ***MMAHC*** | *609342* | chr11: 59,596,741-59,612,974 | | [005142.2](http://www.ncbi.nlm.nih.gov/entrez/viewer.fcgi?val=NM_005142.2) | | 1434 | | 0 | 89.7 | 0 | 99.8 | - | - | | - | - | - |  |
| *CD320* | ***MMAHC*** | *606475* | chr19: 8,367,011-8,373,240 | | [016579.3](http://www.ncbi.nlm.nih.gov/entrez/viewer.fcgi?val=NM_016579.3) | | 949 | | 0 | 116.5 | 0 | 99.9 | - | - | | - | - | - |  |
| *ABCD4* | ***MMAHC*** | *603214* | chr14: 74,752,126-74,769,759 | | [005050.3](http://www.ncbi.nlm.nih.gov/entrez/viewer.fcgi?val=NM_005050.3) | | 2198 | | 0 | 94.6 | 0.2 | 99.7 | - | - | | - | - | - |  |
| *TCN1* | ***MMAHC*** | *189905* | chr11: 59,620,273-59,634,048 | | [001062.3](http://www.ncbi.nlm.nih.gov/entrez/viewer.fcgi?val=NM_001062.3) | | 1482 | | 0 | 83.5 | 0.5 | 98.8 | - | - | | - | - | - |  |
| *AMN* | ***MMAHC*** | *605799* | chr14: 103,388,993-103,399,933 | | [030943.3](http://www.ncbi.nlm.nih.gov/entrez/viewer.fcgi?val=NM_030943.3) | | 1602 | | 0 | 76.1 | 0.1 | 99.3 | - | | - | - | - | - |  |
| *CUBN* | ***MMAHC*** | *602997* | chr10: 16,865,963-17,171,830 | | [001081.3](http://www.ncbi.nlm.nih.gov/entrez/viewer.fcgi?val=NM_001081.3) | | 12212 | | 0 | 77.4 | 0.8 | 98.9 | - | | - | - | - | - |  |
| *CBS* | ***HC*** | *613381* | chr21: 44,473,301-44,497,053 | | [000071.2](http://www.ncbi.nlm.nih.gov/entrez/viewer.fcgi?val=NM_000071.2) | | 1956 | | 0 | 120.4 | 0.2 | 99.6 | 3325 | | 0.8 | 252.9 | 1.5 | 98.3 |  |
| *MTR* | ***HC*** | *156570* | chr1: 236,958,610-237,067,281 | | [000254.2](http://www.ncbi.nlm.nih.gov/entrez/viewer.fcgi?val=NM_000254.2) | | 4458 | | 0 | 83.2 | 0.4 | 99.2 | 10558 | | 0 | 239.4 | 0.1 | 99.9 |  |
| *MTRR* | ***HC*** | *602568* | chr5: 7,851,299-7,906,138 | | [002454.2](http://www.ncbi.nlm.nih.gov/entrez/viewer.fcgi?val=NM_002454.2) | | 2478 | | 0 | 90.9 | 0.7 | 99.0 | 3384 | | 0 | 178.3 | 0 | 100 |  |
| *MTHFR* | ***HC*** | *607093* | chr1: 11,845,780-11,866,977 | | [005957.4](http://www.ncbi.nlm.nih.gov/entrez/viewer.fcgi?val=NM_005957.4) | | 2191 | | 0 | 87.1 | 0.1 | 99.6 | 7150 | | 0 | 323.5 | 0 | 100 |  |
| *MMADHC* | ***HC*** | *611935* | chr2: 150,426,148-150,444,330 | | [015702.2](http://www.ncbi.nlm.nih.gov/entrez/viewer.fcgi?val=NM_015702.2) | | 1031 | | 0 | 86.6 | 0.5 | 99.3 | 1437 | | 0 | 111.2 | 0.1 | 99.4 |  |
| *MTHFD1* | ***HC*** | *172460* | chr14: 64,854,749-64,926,722 | | [005956.3](http://www.ncbi.nlm.nih.gov/entrez/viewer.fcgi?val=NM_005956.3) | | 3348 | | 0 | 78.9 | 0.2 | 99.5 | - | | - | - | - | - |  |
| *ACSF3* | ***CMAMMA*** | *614245* | chr16: 89,154,783-89,222,254 | | [174917.4](http://www.ncbi.nlm.nih.gov/entrez/viewer.fcgi?val=NM_174917.4) | | 4469 | | 0 | 94.8 | 0.7 | 99.0 | - | | - | - | - | - |  |
